# Closed-Form Capacity Reliability Analysis of Multiuser MIMO System in the Presence of Generalized Multipath Fading

**DOI:** 10.3390/s23042289

**Published:** 2023-02-18

**Authors:** Aleksey S. Gvozdarev, Aleksandra M. Alishchuk, Marina A. Kazakova

**Affiliations:** Department of Intelligent Radiophysical Information Systems (IRIS), P.G. Demidov Yaroslavl State University, 150003 Yaroslavl, Russia

**Keywords:** fading, channel, MIMO, Nakagami-m, capacity, higher-order statistics, 89.70.+c, 84.40.Ua, 94A15, 94A40

## Abstract

This research studies the problem of a joint capacity/capacity reliability analysis of the multiuser multi-input multioutput (MIMO) system functioning in the presence of generalized multipath fading. The study presents the derived results of the closed-form analytical statistical description of the ergodic sum-rate capacity, the capacity reliability and the capacity’s higher-order statistics in the case of complex Nakagami-m distributed channel transmission coefficients. A numerical verification of the derived expressions was performed, and it demonstrated excellent correspondence with the simulation. The system performance was evaluated with the help of a numerical analysis of the joint first- and second-order statistics description, depending on the channel and system parameters. The results demonstrated several peculiarities, e.g., the existence of a specific extremum of the capacity reliability for small-sized MIMO systems, its opposite behavior (in terms of the varying number of antenna elements) for heavy and light fading, and the existing asymptotic regions of the system and the channel parameters.

## 1. Introduction

Multiple Input Multiple Output (MIMO) systems has become an intrinsic technological solution for the most modern communication systems [1]. Moreover, the increasing demands for higher throughput (i.e., the increasing utilized spectral bandwidth) and greater connectivity (i.e., the increasing number of communicating devices) extend it to a multiuser massive-MIMO modification [2,3]. In this situation, the forecast of the overall link quality greatly depends on the communicating systems’ description accuracy, including such effects as the transmit/receive antenna correlation [4,5], multipath fading propagation [6], and the specifically employed signal processing strategy. The resultant decision about the need for system parameters adaptation is drawn based on the performance metrics that are being used: error rates, security performance, outage, capacity, etc.

One of the possible descriptions of MIMO systems relies on the higher-order statistics of the channel capacity (C-HOS) [7,8,9,10,11,12,13,14,15]. It results from the fact that under the channel’s random fluctuations, the instantaneous capacity deviates from its stationary value. The rate and magnitude of those deviations may cause sufficient distortions of the overall system performance quality. C-HOS have been intensively studied for various fading channels, diversity reception techniques and specific system configurations: generalized-gamma fading with equal gain (EGC) combiner [14], κ–μ shadowed [15] and η–μ [12] fading with maximum ratio combiner (MRC) and spectrum aggregation by MRC with κ–μ and κ–μ shadowed fading [13].

By now, one of the mainstream approaches in C-HOS calculation is based on the moment-generating function (MGF) application, rather than on the probability density function. First proposed in [8], it was further elaborated in [9,10,11,15] to yield results for specific systems under investigation including the expression of C-HOS in the asymptotic regime for the case of generalized fading (see [10]).

Although the third and the fourth C-HOS (i.e., skewness and kurtosis) did not find wide application, the second moment of the channel capacity (amount of dispersion (AoD)) and its complement (capacity reliability (CR)) were proven to be important indicative factors of the performance quality. Thus, they should be taken into consideration when analyzing MIMO system [12,13,15] functioning in the presence of fading.

Regarding the channel model, it must be stressed out that in the cases of the latest existing and future promising communication systems (for example, 5G, WiFi-7) [16,17] the contraction of the coverage area (especially in urban scenario and inside the buildings) leads to a discrepancy between the practical channel measurements and the initial assumption of the Gaussianity of channel-coefficient in-phase/quadrature components. This, in turn, results in the problem of the overestimation/underestimation of the system’s overall performance, thus leading to incorrect decisions about the deployed strategy (i.e., signal processing, power allocation, etc.). In such a situation, more involved models can be employed (although with a loss of the potential for a closed-form analytical description) [6,18].

On the other hand, as it was demonstrated in [19], the performance of modern communication systems, especially of those employing MIMO technology, is highly sensitive to the phase distribution of the wireless channel’s transmission coefficients, which is generally disregarded in most of the models.

One of the possible alternatives (assumed herein) that can handle both of those problems is the generalization of the well-known Nakagami-m distribution [20], i.e., the complex Nakagami-m model [21,22]. Delivering a compromise between the generality of physical scenarios’ description and analytical tractability, it operates with a signal’s instantaneous values, opposite to the classical one that deals only with the envelope, thus delivering a greater versatility in the ways the phase statistics are accounted.

Initially proposed in [21], it has been widely applied to various problems of wireless communications. For example, in [19] it was used to demonstrate that the knowledge of the phase distribution of the channel’s transmission coefficients significantly affects the performance of spatial multiplexing schemes in MIMO communication systems. In [23], marginal and joint moments of the complex Nakagami-m random variable envelope and phase were obtained and applied to the problem of a random mixture decomposition for the simplification and generalization of bit error probability expressions for the PSK modulation over an amplify-and-forward relay channel. The problem of complex Nakagami-m random variable generation for channel modelling was extensively discussed: in [24] a simulator for both the phase and the envelope with arbitrary parameters was proposed; in [25], it was further improved and included the prespecified temporal autocorrelation function; and in [26], a relation between the Nakagami-m, Gaussian and gamma distributions was exploited to propose a simplified method of physical channel coefficients generation. In [27], a complex Nakagami-m fading model was used to analyze an average bit error rate of the quadrature spatial modulation of a MIMO system. The results were further improved in [28] with the proposed modified generalized quadrature spatial modulation and its reduced codebook version, which were analyzed for the same channel. The OFDM system performance (in terms of the bit error rate of the BPSK modulation) for the case of complex Nakagami-m fading with both uniform and nonuniform phase distributions was analyzed in [4]. In [29], the assumed model was used to assess the communication assistance via reconfigurable intelligent surfaces in terms of the bit error rate, outage probability and ergodic capacity. A performance analysis of the communication systems with the low-resolution analog-to-digital converters (ADC) functioning in the presence of the complex Nakagami-m fading was carried in [30] in terms of the outage probability and the total sum-rate capacity of the MIMO system and in [31,32], in terms of the energy distortion coefficient for the energy-based signal detection.

It must be pointed out that in most modern communications systems, multiuser scenarios are assumed. This means that specific care is needed in the choice of the user’s signal detection algorithms, since it impacts the overall system performance measured, for example, in terms of the ergodic capacity (adopted in the submission) [33]. A classical compromising choice for MIMO systems proposes that the receiver employs the zero-forcing (ZF) processing strategy, which is examined in this research.

One must note that MIMO technology itself is an umbrella-type technology, i.e., it includes various branches that are identified by specific signal precoding/decoding schemes and transmitting/receiving circuitry management. The latter one gave rise to the so-called spatial modulation (SM) [34,35] (generalized spatial modulation (GSM)), which helps to minimize the number of transmitting/receiving radiofrequency (RF) chains and, at the same time, increase the spectral efficiency, for example, generalized quadrature SM [36]. Although for high dimensional MIMO (e.g., more than 8×8), it demonstrates superiority in terms of system capacity over the classical spatial multiplexing [37], it still leaves room for signal precoding/decoding at the stage of antenna management, for example, zero-forcing processing, as one of the prevalent algorithms (see, [38,39]). Despite the very promising features of GSM, it is not widely applied in modern and ad hoc systems, not least due to the complex description and performance analysis; for instance, its closed-form capacity description in the presence of fading is still an open problem and to the best of one’s knowledge, only upper and lower bounds for the simple fading scenarios exist (see [40,41]). Moreover, since most modern communication standards adopt a moderate number of antenna elements, the adopted herein model does not assume GMS, although the obtained results can be further modified by taking into account RF circuitry management.

The analysis of the complex Nakagami-m fading channel’s ergodic capacity has been taken into account in the technical literature several times. As a matter of fact, all the proposed solutions fall into one of three categories: the ones that rely on the random matrix theory methods (applied to the MIMO system/correlation matrix), the ones that imply some limiting properties or asymptotic behavior (yielding upper/lower bounds) or those that are tied to some sort of approximations.

The first approach (see [42]), for the case of a uniform power allocation strategy with no diversity reception, allows one to derive the closed-form expression for the ergodic capacity of the 2×2 and 2×3 MIMO systems in terms of the joint eigenvalue density function of the matrix $W=H^{†}H$ (here, H is the channel matrix). Apparently, due to analytical overcomplication, this approach fails to deliver a closed-form expression for higher dimensions. In [43], the authors assessed the problem via copula theory and presented analytic results for the MIMO 2×2 system. In [44], a Coulomb gas analogy was assumed to derive the approximated cumulative density function of the largest eigenvalue of the matrix W, which, as the authors stated, could be further employed to bound the MIMO system’s performance, although no further improvements were done.

Within the second approach, the authors in [45] obtained the upper bound of the ergodic capacity for the uncorrelated MIMO channel in general (i.e., without specifying a signal processing strategy), and further elaborated the results in [46] with the same channel but including the minimum mean-square error (MMSE) and zero-forcing processing, although limiting the results to only two receivers. The problem of this approach is that, as it was pointed out in [47], for a complex Nakagami-m channel even in the case of ZF beamforming, the presence of correlation across the data streams impedes the derivation of the closed-form expression for the ergodic capacity.

The third approach mostly relies upon the assumption that at some stage of the derivations, the complex closed-form expressions can be efficiently approximated by some easier ones. For example, in [48], it was demonstrated that for uncorrelated Rayleigh channels in the case of power-normalized ZF processing, the output signal-to-noise ratio (SNR) followed the Fisher–Snedecor distribution [49], which could be efficiently approximated by a $\chi^{2}$ distribution (see [50]), and in the general case, the $\chi^{2}$ distribution (see [51,52]) was the closed-form solution. Specifically for the complex Nakagami-m channel, in [53], for the maximum ratio combining receiver, the output SNR per stream was approximated with a more general distribution, the gamma distribution (as it was proposed in [54]), to derive the average spectral efficiency. The same approach was used in [30] for the capacity prediction without antenna correlation but with a specific focus on low-resolution ADCs. The extensive numeric simulation performed later (see [55]) helped to establish a simple per-stream SNR for ZF processing in the presence of complex Nakagami-m fading.

Although much effort has been applied to the problem, no closed-form solution applicable to multiuser massive MIMO systems exists. Moreover, the problem of the capacity reliability has not been approached.

Thus, motivated by the problem stated above and the mentioned drawbacks of the existing solutions, the proposed research performs a closed-form capacity’s higher-order statistics analysis of the multiuser MIMO system employing the zero-forcing postprocessing and operating in the presence of a multipath fading channel described by the complex Nakagami-m distribution. Moreover, to fill in the gap between the existing results, the closed-form expressions for the sum-rate capacity were derived for the arbitrary system size, based on the gamma approximation of the ZF postprocessing’s signal-to-interference-plus-noise ratio. The major contributions of this work can be summarized as follows:

- The closed-form expressions were derived for: (a) the single-stream and sum-rate capacity’s moment-generating functions; (b) the zero-forcing multiuser MIMO ergodic capacity; (c) the capacity reliability and the amount of dispersion; (d) the general-order capacity statistics.
- A thorough joint analysis of the system capacity and its reliability from all possible channel parameters for different fading scenarios—heavy fading and light fading—was made.
- A pronounced extremum of the capacity reliability for small-sized MIMO systems with respect to the fading Nakagami-m parameter was discovered, and the opposing behavior (depending on the system size) for hyper-Rayleigh and lighter-than-Rayleigh fading conditions was demonstrated.
- The asymptotic parameters’ regions where either the ergodic capacity or capacity reliability are almost insensible to the parameters’ change were identified.

The remainder of the paper is organized as follows: Section 2 provides some preliminary results of the assumed (a) wireless multiuser MIMO system and its description in terms of the MIMO channel matrix including receive/transmit side correlation effects, (b) the statistical description of the employed channel model connecting each pair of transmitting and receiving antennas, (c) the signal processing strategy under consideration and (d) the system performance description in terms of the capacity’s higher-order statistics. Section 3 describes the closed-form expression derivation of the assumed C-HOS. Section 4 presents a thorough numerical analysis of the derived expression depending on various channel and system parameters’ values, and the conclusions are drawn in Section 5.

## 2. General System Description

### 2.1. System Model

This research considered a multiuser MIMO communication system with $N_{T}$ transmitting and $N_{R}$ receiving antenna elements functioning in the presence of a multipath fading propagation. The received signal vector model $\vec{y}$ was mathematically formalized as follows [56]:

(1) $$\vec{y}=H\vec{x}+\vec{n},$$

where $\vec{x}$ is the transmitted signal vector, $\vec{n}$ is the vector of zero-mean circular symmetric additive complex white Gaussian noise (AWGN) with variance $\sigma^{2}$ (i.e., $n_{i}∼\mathrm{CN}\left(0,\sigma^{2}\right)$), and H is the MIMO channel matrix.

For later derivations, it was assumed that H complies with the Kronecker separable model (see, for instance, Section 4.2.2.3 in [57]):

(2) H=ΣR12HwΣT12,

where ΣT12 and ΣR12 are the matrix square roots of the transmitting/receiving side correlation matrices, and matrix $H_{w}$ is composed of the independent complex transmission coefficients (i.e., ${\left[H_{w}\right]}_{i,j}={\dot{h}}_{i,j}$) between any pair of receiving and transmitting antennas [57].

Since it is usually assumed that the correlation decays with the spacing between the interarray antenna elements [56,57], this research considered a widely used exponential correlation model [58] (for both sides) described by the so-called one–step correlation coefficient $\rho_{i,j}$, which quantifies the correlation between the *i*th and *j*th elements:

(3) $${\left[\Sigma \right]}_{i,j}=\left(\begin{array}{ccccc} 1 & \rho & \rho^{2} & \cdots & \rho^{N-1}\\ \;\rho & 1 & \rho & \cdots & \rho^{N-2}\\ \rho^{2} & \rho & 1 & \cdots & \rho^{N-3}\\ \;\cdots & \cdots & \cdots & \cdots & \cdots \\ \rho^{N-1} & \rho^{N-2} & \rho^{N-3} & \cdots & 1 \end{array}\right),$$

where $\rho =\rho_{t}$ if $\Sigma =\Sigma_{T}$ (i.e., the transmitting side is assumed), and $\rho =\rho_{r}$ if $\Sigma =\Sigma_{R}$ (i.e., for the receiving side).

### 2.2. Channel Model

As was mentioned earlier, the majority of the existing results in MIMO communications rely on the assumption that the complex channel transmission coefficients ${\dot{h}}_{i,j}$ are subject to a complex Gaussian distribution [59]. This assumption is generally valid due to the central limit theorem applied to the multitude of (a) propagation paths, connecting any pair of the transmitter and the receiver, and (b) the reflections/multiple-reflection/diffraction of each multipath component. For such scenarios, the statistical properties (e.g., probability density function, cumulative distribution function, moment-generating function, etc.) of the complex channel matrix H are available via the random matrix theory [60,61].

On the other hand, modern communication systems exhibit shrinkage of the coverage area [62] leading to the decrease of the number of multipath components, hence failing to comply with the Gaussian approximation. In this case, a more suitable distribution can be assumed for ${\dot{h}}_{i,j}$.

This research considered the complex transmission coefficient to follow the complex Nakagami-m distribution, which is defined in terms of its envelope ($|\dot{h}|$)/phase ($\Theta =∠\dot{h}$) distributions:

(4a) $$w_{|\dot{h}|}\left(r\right)=\frac{2m^{m}r^{2m-1}}{\Omega^{m}\Gamma \left(m\right)}\exp\left(-\frac{mr^{2}}{\Omega }\right),\;0\leq r<\infty ,$$

(4b) $$w_{\Theta }\left(\theta \right)=\frac{{\Gamma \left(m\right)|\sin2\Theta |}^{m-1}}{2^{m}\Gamma^{2}\left(\frac{m}{2}\right)},\;-\pi \leq \theta \leq \pi ,$$

or, equivalently, in terms of its in-phase $h_{I}=ℜ\left({\dot{h}}_{i,j}\right)$ and quadrature $h_{Q}=ℑ\left({\dot{h}}_{i,j}\right)$ components’ distributions:

(5) $$w_{h_{I,Q}}\left(z\right)=\frac{m^{\frac{m}{2}}{\left|z\right|}^{m-1}}{\Omega^{\frac{m}{2}}\Gamma \left(\frac{m}{2}\right)}\exp\left(-\frac{{m|z|}^{2}}{\Omega }\right),\;-\infty <z<\infty .$$

The model is described in terms of two parameters:

- The mean power of the received signal Ω, i.e., $\Omega =E\{|\dot{h}{|}^{2}\}$;
- The Nakagami fading parameter *m*, which is the inverse of the amount of fading $m=\frac{{\left(E\{|\dot{h}{|}^{2}\}\right)}^{2}}{V\mathrm{ar}\{|\dot{h}{|}^{2}\}}$.

Here, E{·} is the expectation operator, and Var{·} is the variance operator.

The flexibility of this model is mainly contingent on the fact that for certain values of *m*, the expressions (4a)–(5) can be simplified, leading to:

- Rayleigh fading, when m=1 [21];
- Lighter-than-Rayleigh fading, when m>1 (including the uniform distribution on the unit circle [42], i.e., m→∞);
- Hyper-Rayleigh [63,64] (heavier than Rayleigh) fading, when m<1 (including the one-sided Gaussian distribution [21], i.e., $m\to \frac{1}{2}$).

Thus, for the subsequent analysis, when the Nakagami parameter is varied, those three particular cases are addressed specifically.

### 2.3. Signal Processing Model

Since a multiuser system is assumed, to improve the signal-to-noise-plus-interference ratio on the receiving side, specific decoding algorithms of the received signal are usually utilized. Hence, it is important to mention the way the user streams are processed. In this paper, a zero-forcing algorithm was used, for which the output instantaneous SNR for the *k*th user (i.e., $\gamma_{k}$) was defined as:

(6) $$\gamma_{k}=\frac{\bar{\gamma }}{{\left[{\left(H^{†}H\right)}^{-1}\right]}_{k,k}},$$

where $\bar{\gamma }$ is the average input SNR for the *k*th substream, ${\left[\cdot \right]}_{k,k}$ is the *k*th element on the main diagonal, and ${\left(\cdot \right)}^{†}$, ${\left(\cdot \right)}^{-1}$ are the Hermitian conjugation and matrix inversion operators.

### 2.4. Capacity’s Higher-Order Statistics

As mentioned earlier, the main objective of the present research was to study the capacity reliability of the MIMO system, which is the special case of a more general methodology, i.e., the capacity’s higher-order analysis [7,8].

Within such a framework, the channel capacity’s (normalized to unit bandwidth) *n*th order moment is defined as:

(7) $$\bar{C_{\Sigma }^{n}}=E\left\{C_{\Sigma }^{n}\right\}=\sum_{k=1}^{N_{s}}E\left\{\log_{2}^{n}\left(1+\gamma_{k}\right)\right\},$$

where $\gamma_{k}$ is the signal-to-noise-plus-interference ratio for the *k*th substream, and $N_{s}$ is the number of active substreams. Assuming the applied processing (see Section 2.3), one obtains:

(8) $$\bar{C_{\Sigma }^{n}}=\sum_{k=1}^{N_{s}}\int_{0}^{\infty }\log_{2}^{n}\left(1+x\right)w_{\gamma_{k}}\left(x\right)dx,$$

where $w_{\gamma_{k}}\left(x\right)$ is the probability density function of the *k*th substream SNR after ZF postprocessing.

The ergodic capacity ${\bar{C}}_{\Sigma }$ (being the first-order moment, i.e., n=1) was used in this work as a metric, which helped to quantify the performance of the assumed system.

For a deeper analysis of the MIMO system functioning, ${\bar{C}}_{\Sigma }$ can be supplemented by the second-order moment (the amount of dispersion (AoD)) and its complement (the reliability of the capacity (R)):

(9) R=1−AoD,

(10) $$\begin{array}{cccc} AoD & & = & \frac{V\mathrm{ar}\{C_{\Sigma }\}}{E\left\{C_{\Sigma }\right\}}=\frac{E\left\{C_{\Sigma }^{2}\right\}-{\left(E\left\{C_{\Sigma }\right\}\right)}^{2}}{E\left\{C_{\Sigma }\right\}}. \end{array}$$

The amount of dispersion describes the normalized spread of the channel capacity’s stochastic variations, thus it quantifies the distortion in the ergodic capacity per one-bit information transfer [11], whereas the capacity reliability is a complementary metric that defines its stability.

From a practical point of view, it is valuable to attain the maximum achievable capacity of the communication channel, simultaneously providing its minimum distortion. Hence, the evaluation of AoD and CR is a substantial element of the link quality estimation and prediction.

## 3. Derived Analytical Results

As mentioned earlier, there are several possible approaches to the problem of a closed-form MIMO system’s capacity description. Since the non-Gaussianity of fading channel statistics prevented us from conducting a closed-form analysis even in the case of ZF processing (see [47]), the approach based on some type of approximation was assumed herein.

### 3.1. Preliminary Results

At this stage, we relied on the results of a thorough numerical analysis (presented in [55]) that was carried out for numerous fading scenarios and various channel/system parameters. It was demonstrated that for a wide range of parameters, the probability distribution of the per-stream signal-to-noise-plus-interference ratio $\gamma_{k}$ could be efficiently approximated with a gamma distribution. To achieve that, a numerical simulation was performed for the MIMO system and fading channel described in Section 2. Several classes of approximating distributions were assumed, including gamma, $\chi^{2}$, Rayleigh, Rician, etc. The approximation quality was assessed by two criteria: Kolmogorov–Smirnov and Pearson’s $\chi^{2}$. The approximation parameters were estimated via a maximum-likelihood procedure. The resultant approximation was assumed reasonable if both statistical tests were jointly passed with a confidence level greater than 95%. It was found out (see [55]) that the most suitable one was the two-parametric gamma distribution (which corresponded to the existing results [53,54]). Moreover, for a ZF processing, the first parameter (the shape parameter) could be set to unity (e.g., $\gamma_{k}∼\Gamma \left(1,{\hat{\beta }}_{k}\right)$), thus resulting in a single unknown parameter $\beta_{k}$, which depended on the system/channel characteristics, but could be easily estimated via the simple averaging of the measured SNR [49].

### 3.2. Capacity’s Higher-Order Statistics Derivation

#### 3.2.1. Moment-Generating Function Derivation

Based on the proposed approximation (i.e., $\gamma_{k}∼\Gamma \left(1,{\hat{\beta }}_{k}\right)$), the moment-generating function of the sum-rate capacity can be derived. To calculate it, one can notice the monotonicity of the functional relation $C_{k}=\log_{2}\left(1+\gamma_{k}\right)$ between $\gamma_{k}$ and $C_{k}$ (that is, the instantaneous capacity per stream) and apply the classical transformation of random variables:

(11) $$w_{C_{k}}\left(x\right)=w_{\gamma_{k}}\left(f^{-1}\left(x\right)\right)\cdot \left|\frac{df^{-1}\left(x\right)}{dx}\right|,$$

where $f^{-1}\left(\cdot \right)$ is the inverse monotonic transform, i.e., $f^{-1}\left(x\right)=\gamma_{k}=2^{x}-1$. Finally, we obtain:

(12) $$w_{C_{k}}\left(x\right)=2^{x}\ln2w_{\gamma_{k}}\left(2^{x}-1\right)=\frac{2^{x}\ln2}{{\hat{\beta }}_{k}}e^{\frac{1}{{\hat{\beta }}_{k}}}e^{-\frac{2^{x}}{{\hat{\beta }}_{k}}},$$

which is clearly a probability density function of the Gompertz–Makeham distribution [65,66], i.e., $C_{k}\overset{d}{∼}\;\mathrm{GM}\left(\ln2,\frac{1}{{\hat{\beta }}_{k}}\right)$.

Using the definition of the MGF (that is, $M_{C_{k}}\left(s\right)=E\left\{e^{sC_{k}}\right\}$), one gets:

(13) $$M_{C_{k}}\left(s\right)=\int_{0}^{\infty }w_{C_{k}}\left(x\right)e^{xs}dx={{\hat{\beta }}_{k}}^{-1}e^{{{\hat{\beta }}_{k}}^{-1}}E_{-\frac{s}{\ln2}}\left({{\hat{\beta }}_{k}}^{-1}\right),$$

where E(·) is the exponential integral [67]. So the moment-generating function of the sum-rate capacity $C_{\Sigma }$ can be evaluated as:

(14a) $$\begin{array}{cccc} & M_{C_{\Sigma }}\left(s\right) & = & \prod_{k=1}^{N_{R}}M_{C_{k}}\left(s\right)=\prod_{k=1}^{N_{R}}{\hat{\beta }}_{k}^{-1}e^{{\hat{\beta }}_{k}^{-1}}E_{-\frac{s}{\ln2}}\left({\hat{\beta }}_{k}^{-1}\right) \end{array}$$

(14b) $$\begin{array}{cccc} & & = & \left(\prod_{k=1}^{N_{R}}{\hat{\beta }}_{k}^{-\frac{s}{\ln2}}\right)\prod_{k=1}^{N_{R}}U\left(-\frac{s}{\ln2},-\frac{s}{\ln2},\frac{1}{{\hat{\beta }}_{k}}\right) \end{array}$$

(14c) $$\begin{array}{cccc} & & = & \left(\prod_{k=1}^{N_{R}}\frac{1}{{\hat{\beta }}_{k}}\right)\prod_{k=1}^{N_{R}}U\left(1,2+\frac{s}{\ln2},\frac{1}{{\hat{\beta }}_{k}}\right), \end{array}$$

where U(·) is the Tricomi confluent hypergeometric function [67]; the second line (14b) and the third line (14c) are due to the connection of the exponential integral with U(·) and the argument shifting property of the Tricomi function (see [67], Equation (13.6.6):

(15) $$z^{1-a}e^{z}E_{a}\left(z\right)=U\left(a,a,z\right),$$

(16) $$U\left(a,a,z\right)=z^{1-a}U\left(1,2-a,z\right).$$

For the subsequent derivations, Equation (14c) was used. It should be noted that if the probability density function is sought, (14c) can be inverted via Gil-Pelaez’s formula [68] (see, for example, ref. [69,70]), or by using the Laplace transform inversion approach [69]. Although such inversion cannot be performed analytically, there is a wide range of approaches to perform it numerically with high accuracy [71].

#### 3.2.2. Ergodic Capacity Derivation

The obtained expression for the sum-rate capacity MGF (14c) helps to calculate the ergodic capacity of the system by taking a derivative at s=0, i.e.,

(17) $${\bar{C}}_{\Sigma }={\left.\frac{dM_{C_{\Sigma }}\left(s\right)}{ds}\right|}_{s=0}.$$

The application of the general Leibniz’s differentiation rule yields:

(18a) $$\begin{array}{cc} \frac{dM_{C_{\Sigma }}\left(s\right)}{ds} & =\left(\prod_{k=1}^{N_{R}}\frac{1}{{\hat{\beta }}_{k}}\right)\sum_{j=1}^{N_{R}}\left(\frac{d}{ds}U\left(1,2+\frac{s}{\ln2},\frac{1}{{\hat{\beta }}_{j}}\right)\right)\prod_{\begin{array}{c} k=1\\ k\neq j \end{array}}^{N_{R}}U\left(1,2+\frac{s}{\ln2},\frac{1}{{\hat{\beta }}_{k}}\right) \end{array}$$

(18b) $$\begin{array}{cccc} & & = & \left(\prod_{k=1}^{N_{R}}\frac{1}{{\hat{\beta }}_{k}}\right)\prod_{k=1}^{N_{R}}U\left(1,2+\frac{s}{\ln2},\frac{1}{{\hat{\beta }}_{k}}\right)\sum_{k=1}^{N_{R}}\frac{\frac{d}{ds}U\left(1,2+\frac{s}{\ln2},\frac{1}{{\hat{\beta }}_{k}}\right)}{U\left(1,2+\frac{s}{\ln2},\frac{1}{{\hat{\beta }}_{k}}\right)} \end{array}$$

(18c) $$\begin{array}{cccc} & & = & M_{C_{\Sigma }}\left(s\right)\sum_{k=1}^{N_{R}}\frac{\frac{d}{ds}U\left(1,2+\frac{s}{\ln2},\frac{1}{{\hat{\beta }}_{k}}\right)}{U\left(1,2+\frac{s}{\ln2},\frac{1}{{\hat{\beta }}_{k}}\right)}. \end{array}$$

Taking the derivative of the Tricomi function with respect to the parameter *s*, we get:

(19) $$\begin{array}{c} \frac{d}{ds}U\left(1,2+\frac{s}{\ln2},\frac{1}{{\hat{\beta }}_{k}}\right)=\frac{{\hat{\beta }}_{k}}{\ln2}e^{\frac{1}{{\hat{\beta }}_{k}}}G_{2,3}^{\;3,0}\;\left(\left.\begin{array}{c} 1-\frac{s}{\ln2},1-\frac{s}{\ln2}\\ 1,-\frac{s}{\ln2},-\frac{s}{\ln2} \end{array}\;\right|\;\frac{1}{{\hat{\beta }}_{k}}\right), \end{array}$$

where $G_{2,3}^{\;3,0}\left(\cdot \right)$ is the Meijer G-function [67]. It can be noticed that

(20) $$M_{C_{\Sigma }}\left(0\right)=1,$$

(21) $$U\left(1,2,{\hat{\beta }}_{k}^{-1}\right)={\hat{\beta }}_{k},$$

(22) $$G_{2,3}^{\;3,0}\;\left(\left.\begin{array}{c} 1,1\\ 1,0,0 \end{array}\;\right|\;\frac{1}{{\hat{\beta }}_{k}}\right)=G_{1,2}^{\;2,0}\;\left(\left.\begin{array}{c} 1\\ 0,0 \end{array}\;\right|\;\frac{1}{{\hat{\beta }}_{k}}\right)=\Gamma \left(0,\frac{1}{{\hat{\beta }}_{k}}\right),$$

where Γ(·) is the upper incomplete gamma-function [67], and the third equality holds true due to Equation (16.19.3) in [67].

Finally, the derived closed-form expression for the ergodic capacity can be formulated as:

(23) $${\bar{C}}_{\Sigma }=\frac{1}{\ln2}\sum_{k=1}^{N_{R}}e^{\frac{1}{{\hat{\beta }}_{k}}}\Gamma \left(0,\frac{1}{{\hat{\beta }}_{k}}\right).$$

#### 3.2.3. Second-Order Capacity Statistics: The Amount of Dispersion, Capacity Reliability

Since the main focus of this research is the MIMO systems’ capacity reliability analysis, according to its definition (9) and (10), the second-order capacity statistics is necessary:

(24) $$\bar{C_{\Sigma }^{2}}={\left.\frac{d^{2}M_{C_{\Sigma }}\left(s\right)}{ds^{2}}\right|}_{s=0}.$$

To this extent, one can make use of (18c):

(25a) $$\begin{array}{cccc} \frac{d^{2}M_{C_{\Sigma }}\left(s\right)}{ds^{2}} & & = & \frac{d}{ds}\left(M_{C_{\Sigma }}\left(s\right)\sum_{k=1}^{N_{R}}\frac{\frac{d}{ds}U\left(1,2+\frac{s}{\ln2},\frac{1}{{\hat{\beta }}_{k}}\right)}{U\left(1,2+\frac{s}{\ln2},\frac{1}{{\hat{\beta }}_{k}}\right)}\right) \end{array}$$

(25b) $$\begin{array}{cccc} & & = & \frac{dM_{C_{\Sigma }}\left(s\right)}{ds}\sum_{k=1}^{N_{R}}\frac{\frac{d}{ds}U\left(1,2+\frac{s}{\ln2},\frac{1}{{\hat{\beta }}_{k}}\right)}{U\left(1,2+\frac{s}{\ln2},\frac{1}{{\hat{\beta }}_{k}}\right)}+\frac{d}{ds}\left(\sum_{k=1}^{N_{R}}\frac{\frac{d}{ds}U\left(1,2+\frac{s}{\ln2},\frac{1}{{\hat{\beta }}_{k}}\right)}{U\left(1,2+\frac{s}{\ln2},\frac{1}{{\hat{\beta }}_{k}}\right)}.\right) \end{array}$$

First, note that

(26) $$\begin{array}{cccc} \frac{dM_{C_{\Sigma }}\left(s\right)}{ds} & & = & {\bar{C}}_{\Sigma }, \end{array}$$

(27) $$\begin{array}{cccc} {\left.\sum_{k=1}^{N_{R}}\frac{\frac{d}{ds}U\left(1,2+\frac{s}{\ln2},\frac{1}{{\hat{\beta }}_{k}}\right)}{U\left(1,2+\frac{s}{\ln2},\frac{1}{{\hat{\beta }}_{k}}\right)}\right|}_{s=0} & & = & {\bar{C}}_{\Sigma }. \end{array}$$

The second term in (25b) evaluated at s=0 can be simplified using (19), (21) and (22) and

(28) $${\left.\frac{d^{2}}{d^{2}s}U\left(1,2+\frac{s}{\ln2},\frac{1}{{\hat{\beta }}_{k}}\right)\right|}_{s=0}=\frac{2{\hat{\beta }}_{k}}{\ln^{2}2}e^{\frac{1}{{\hat{\beta }}_{k}}}G_{2,3}^{\;3,0}\;\left(\left.\begin{array}{c} 1,1\\ 0,0,0 \end{array}\;\right|\;\frac{1}{{\hat{\beta }}_{k}}\right).$$

Finally, the second raw moment of the capacity of the assumed MU-MIMO system is given by

(29) $$\bar{C_{\Sigma }^{2}}={\left({\bar{C}}_{\Sigma }\right)}^{2}+\sum_{k=1}^{N_{R}}\frac{e^{\frac{1}{{\hat{\beta }}_{k}}}}{\ln^{2}2}\left[2G_{2,3}^{\;3,0}\;\left(\left.\begin{array}{c} 1,1\\ 0,0,0 \end{array}\;\right|\;\frac{1}{{\hat{\beta }}_{k}}\right)-e^{\frac{1}{{\hat{\beta }}_{k}}}\Gamma^{2}\left(0,\frac{1}{{\hat{\beta }}_{k}}\right)\right].$$

This helps to evaluate the amount of dispersion:

(30) $$AoD=\frac{\sum_{k=1}^{N_{R}}e^{\frac{1}{{\hat{\beta }}_{k}}}\left[2G_{2,3}^{\;3,0}\;\left(\left.\begin{array}{c} 1,1\\ 0,0,0 \end{array}\;\right|\;\frac{1}{{\hat{\beta }}_{k}}\right)-e^{\frac{1}{{\hat{\beta }}_{k}}}\Gamma^{2}\left(0,\frac{1}{{\hat{\beta }}_{k}}\right)\right]}{\ln2\sum_{k=1}^{N_{R}}e^{\frac{1}{{\hat{\beta }}_{k}}}\Gamma \left(0,\frac{1}{{\hat{\beta }}_{k}}\right)}.$$

A further simplification can be obtained by reducing the Meijer G-function to a simpler one, thus yielding the final closed-form expression for the capacity reliability, that was used for our further analysis:

(31) $$\begin{array}{c} R=1-{\left(\ln2\sum_{k=1}^{N_{R}}e^{\frac{1}{{\hat{\beta }}_{k}}}\Gamma \left(0,\frac{1}{{\hat{\beta }}_{k}}\right)\right)}^{-1}\sum_{k=1}^{N_{R}}e^{\frac{1}{{\hat{\beta }}_{k}}}\left[\frac{2}{{\hat{\beta }}_{k}}{\;}_{3}F_{3}\left(1,1,1;2,2,2;-\frac{1}{{\hat{\beta }}_{k}}\right)+\right.\\ \left.+\ln^{2}\left({\hat{\beta }}_{k}\right)-2C_{e}\ln\left({\hat{\beta }}_{k}\right)+C_{e}+\frac{\pi^{2}}{6}-e^{\frac{1}{{\hat{\beta }}_{k}}}\Gamma^{2}\left(0,\frac{1}{{\hat{\beta }}_{k}}\right)\right], \end{array}$$

where $C_{e}$ is the Euler–Mascheroni constant [67], and ${}_{3}F_{3}\left(\cdot \right)$ is the generalized univariate hypergeometric function [67].

#### 3.2.4. Further Generalization: *n*th Order Capacity Statistics

To derive the generalized expression, first, let us rewrite Equation (18c) in a compact form:

(32) $$\begin{array}{cccc} \frac{dM_{C_{\Sigma }}\left(s\right)}{ds} & & = & M_{C_{\Sigma }}\left(s\right)g\left(s\right), \end{array}$$

where g(s) denotes the sum of logarithmic derivatives of Tricomi functions.

In case of such a notation, the *n*th order capacity statistics is expressed as

(33a) $$\begin{array}{cccc} \bar{C_{\Sigma }^{n}}={\left.\frac{d^{n}M_{C_{\Sigma }}\left(s\right)}{ds^{n}}\right|}_{s=0} & & = & {\left.\frac{d^{n-1}}{ds^{n-1}}\left(M_{C_{\Sigma }}\left(s\right)g\left(s\right)\right)\right|}_{s=0} \end{array}$$

(33b) =∑j=0n−1n−1jdjMCΣ(s)dsjs=0dn−j−1g(s)dsn−j−1s=0

(33c) =∑j=0n−1n−1jCΣj¯dn−j−1g(s)dsn−j−1s=0,

where the second line is obtained via the general Leibniz rule, and ·· is the binomial coefficient [67].

Using the result from [72] (see Chapter I, page 40) the *r*th order logarithmic derivative of a Tricomi function can be rewritten as the following r+1-order determinant:

(34) drg(s)dsr∝drdsrdU(·)dsU(·)=(−1)rUr+1(·)U′(·)U(·)0…0U″(·)U′(·)U(·)…0U‴(·)U″(·)2U′(·)…0………⋱…U(r)(·)U(r−1)(·)r−11U(r−2)(·)⋱U(·)U(r+1)(·)U(r)(·)r1U(r−1)(·)⋱rr−1U′(·),

where a shorthand notation $U^{\left(\cdot \right)}\left(\cdot \right)$ is used instead of the derivative of the Tricomi function $U\left(1,2+\frac{s}{\ln2},\frac{1}{{\hat{\beta }}_{k}}\right)$.

It was shown that (34) can be efficiently reorganized (see Equation (1.5) in [73]), and thus the derivative of g(s) in (33c) can be reformulated in terms of a determinant of a Heisenberg matrix W (size (n−j)×(n−j)):

(35) $$\frac{d^{n-j-1}g\left(s\right)}{ds^{n-j-1}}=\frac{{\left(-1\right)}^{n-j-1}}{U^{n-j}\left(1,2+\frac{s}{\ln2},\frac{1}{{\hat{\beta }}_{k}}\right)}\left|W_{\left(n-j)\times (n-j\right)}\left(s\right)\right|,$$

which is composed of a column matrix $P_{(n-j)\times 1}\left(s\right)$ and a rectangular matrix $Q_{\left(n-j)\times (n-j-1\right)}\left(s\right)$ (i.e., $\left(P_{(n-j)\times 1}\left(s\right),Q_{\left(n-j)\times (n-j-1\right)}\left(s\right)\right)$) with elements:

(36) $$P_{r,1}\left(s\right)=\frac{d^{r}}{ds^{r}}U\left(1,2+\frac{s}{\ln2},\frac{1}{{\hat{\beta }}_{k}}\right)\;1\leq r\leq \left(n-j\right),$$

(37) Qr,l(s)=r−1l−1dr−ldsr−lU1,2+sln2,1β^kif(r−l)≥0,1≤r≤(n−j),0if(r−l)<0,1≤l≤(n−j−1).

To finalize the result, one can notice that the derivatives of the Tricomi function in (36) and (37) can be obtained in a closed form:

(38) $$\frac{d^{r}}{ds^{r}}U\left(1,2+\frac{s}{\ln2},\frac{1}{{\hat{\beta }}_{k}}\right)=\frac{r!{\hat{\beta }}_{k}e^{\frac{1}{{\hat{\beta }}_{k}}}}{\ln^{r}2}G_{r+1,r+2}^{\;r+2,0}\;\left(\left.\begin{array}{c} 1-\frac{s}{\ln2},\ldots 1-\frac{s}{\ln2}\\ 1,-\frac{s}{\ln2},\ldots -\frac{s}{\ln2} \end{array}\;\right|\;\frac{1}{{\hat{\beta }}_{k}}\right).$$

Since their values at s=0 are sought, the formula for lowering the order of the Meijer G-function can be efficiently used (see Equation (8.2.2.8) in [74]), yielding:

(39) $${\left.\frac{d^{r}}{ds^{r}}U\left(1,2+\frac{s}{\ln2},\frac{1}{{\hat{\beta }}_{k}}\right)\right|}_{s=0}=\frac{r!{\hat{\beta }}_{k}e^{\frac{1}{{\hat{\beta }}_{k}}}}{\ln^{r}2}G_{r,r+1}^{\;r+1,0}\;\left(\left.\begin{array}{c} 1,\ldots 1\\ 0,\ldots 0 \end{array}\;\right|\;\frac{1}{{\hat{\beta }}_{k}}\right).$$

The resultant closed-form expression for the *n*th order capacity statistics is given by:

(40) CΣn¯=∑j=0n−1n−1j(−1)n−j−1CΣj¯∑k=1NRW(0)β^kn−j,

where the elements of the matrix W(0) are evaluated according to expressions (36), (37) and (39).

To the best of the authors’ knowledge, the closed-form capacity’s higher-order statistics analysis is absent in the current technical literature, and the results derived in Section 3.2 are novel and have not been reported previously.

## 4. Simulation and Results

To verify the correctness of the derived expressions and perform a higher-order capacity analysis, a numeric simulation was carried out.

The simulation was executed in accordance with the general theory described in Section 2 (see Section 2.1 for the system model description, Section 2.2 for the channel model, Section 2.3 for the correlation model and Section 2.4 for the signal processing model).

The assumed system and channel parameter values are presented in Table 1.

To this extent, several important notes should be pointed out:

- The one-step correlation coefficient was chosen in such a way (i.e., not exceeding 0.5) as to comply with the existing results for the bordered correlation matrices (see [55]), where the maximum possible ρ (that yielded physically meaningful results, i.e., positive-definite system correlation matrices) was 0.5 (see [75]).
- The $N_{s}$ was chosen in such a way as to cover the case when all the users are active (maximum number of active eigenstreams).
- The mean power of the received signal in the communication channel (Ω) was set to unity, since it could be efficiently recalculated into the average signal-to-noise ratio, which was swept by the range of 0…50dB.
- The fading parameter *m* was upper-bounded by m=5 since numerous research works have demonstrated that this value can be assumed as “almost asymptotic”, which leads to the fact that its increase does not induce significant changes in the result.
- The parameter *m* was set in such a way as to account for hyper-Rayleigh fading (i.e., 0.5≤m<1), Rayleigh (m=1) and lighter-than-Rayleigh fading (m>1).

**Figure 1:**
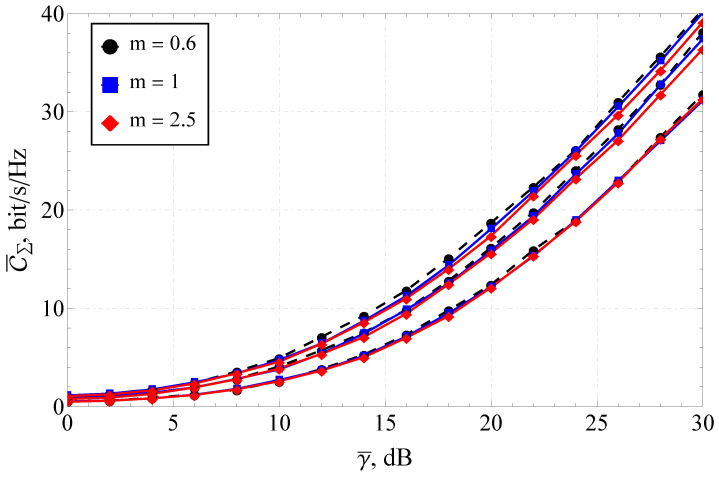
Ergodic capacity ${\bar{C}}_{\Sigma }$ of the 8×8 MIMO system for various *m* and correlation coefficients.

**Figure 2:**
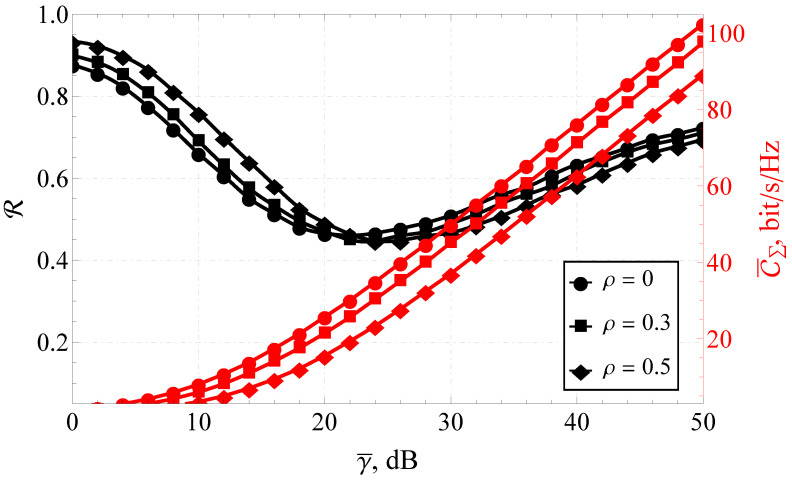
The joint R–${\bar{C}}_{\Sigma }$ performance of the 8×8 for a variable $\bar{\gamma }$.

**Figure 3:**
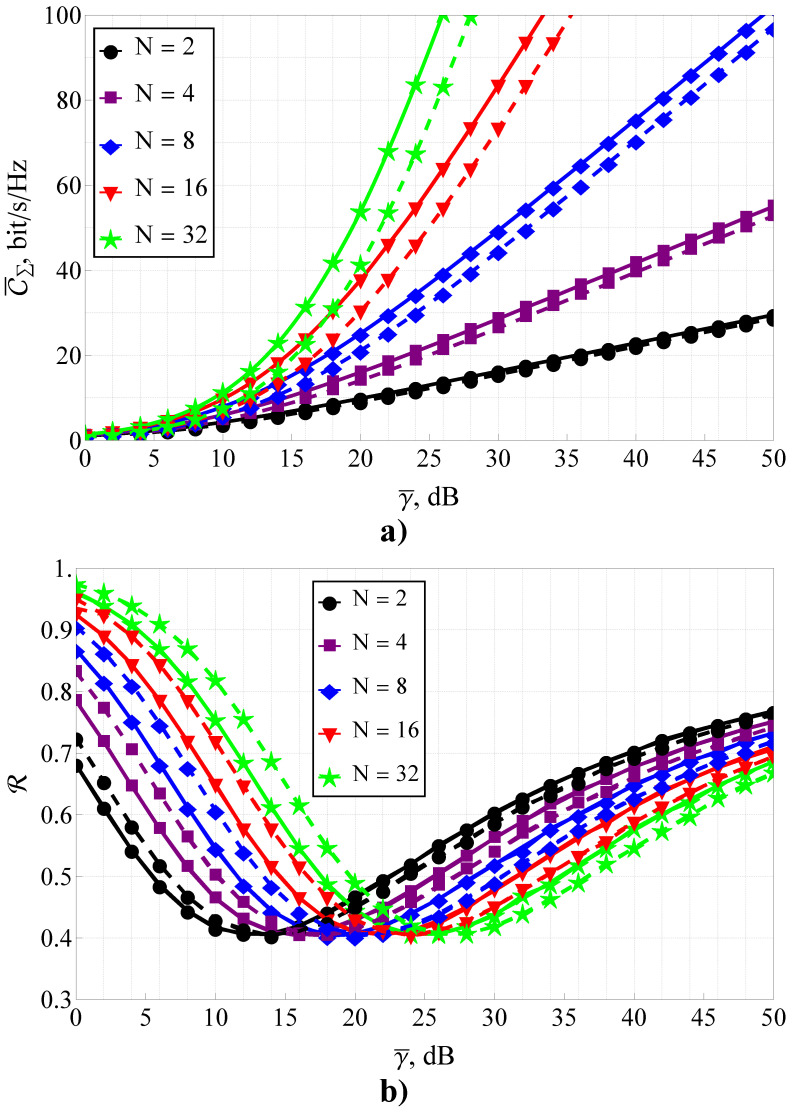
${\bar{C}}_{\Sigma }$ (subfigure (**a**)) and R (subfigure (**b**)) for variable $\bar{\gamma }$ and different MIMO system size N×N: solid lines depict the case of ρ=0.1, dashed lines depict the case of ρ=0.5.

**Figure 4:**
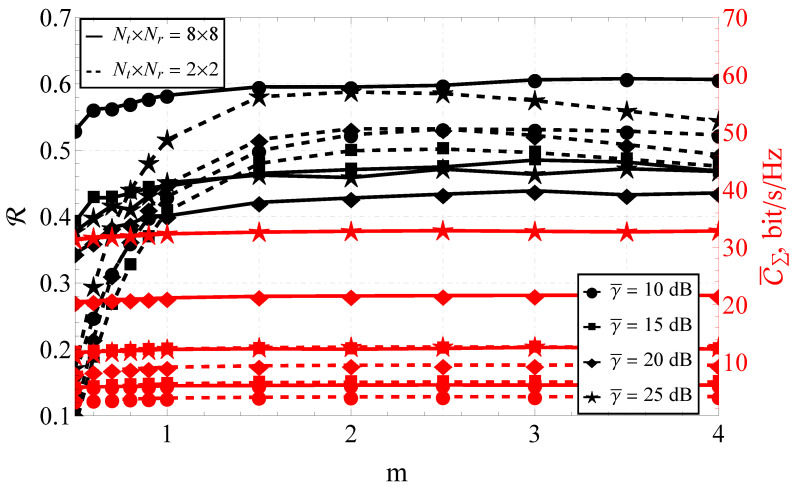
The joint R–${\bar{C}}_{\Sigma }$ performance of the 8×8 and 2×2 MIMO systems for various *m*’s and different $\bar{\gamma }$’s.

**Figure 5:**
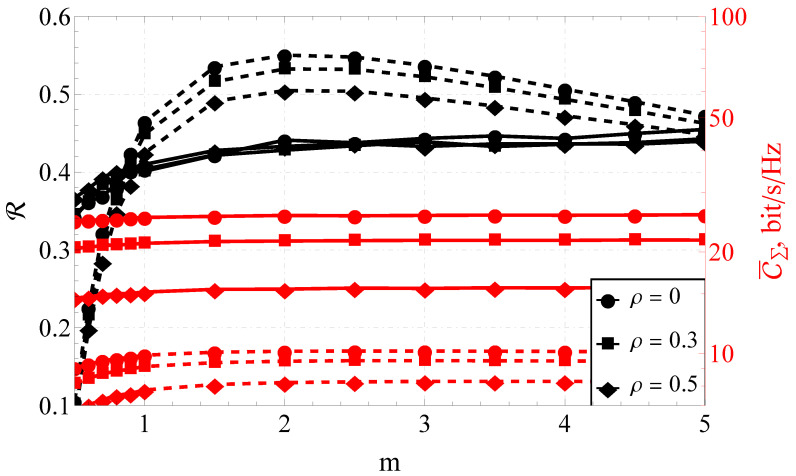
The joint R–${\bar{C}}_{\Sigma }$ performance of the 8×8 and 2×2 MIMO systems for various *m*’s, $\bar{\gamma }=20$ dB, and different ρ’s.

**Figure 6:**
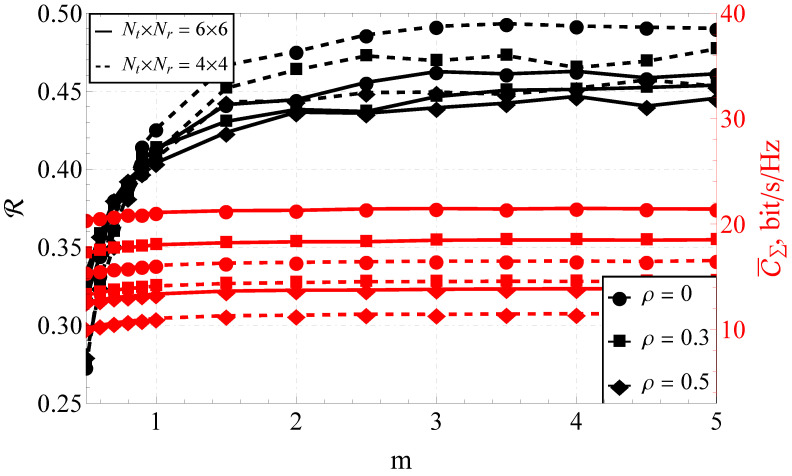
The joint R–${\bar{C}}_{\Sigma }$ performance of the 6×6 and 4×4 MIMO systems for various *m*’s, $\bar{\gamma }=20$ dB, and different ρ’s.

**Figure 7:**
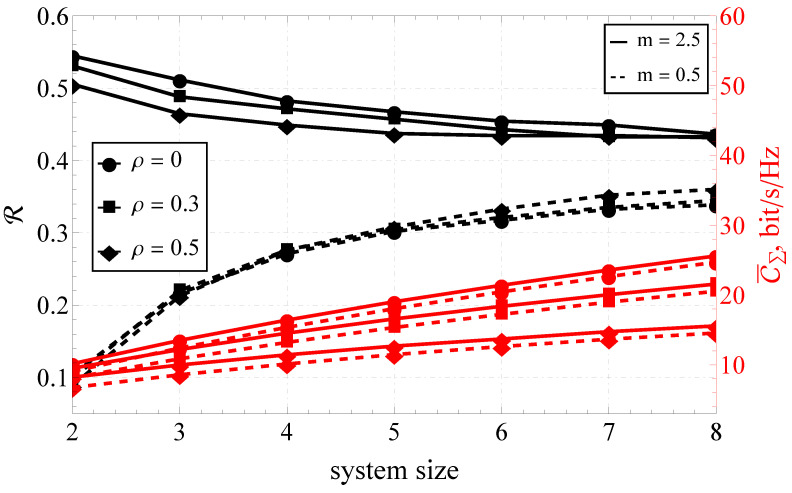
The joint R–${\bar{C}}_{\Sigma }$ performance of the variable-size MIMO system and different ρ’s.

**Figure 8:**
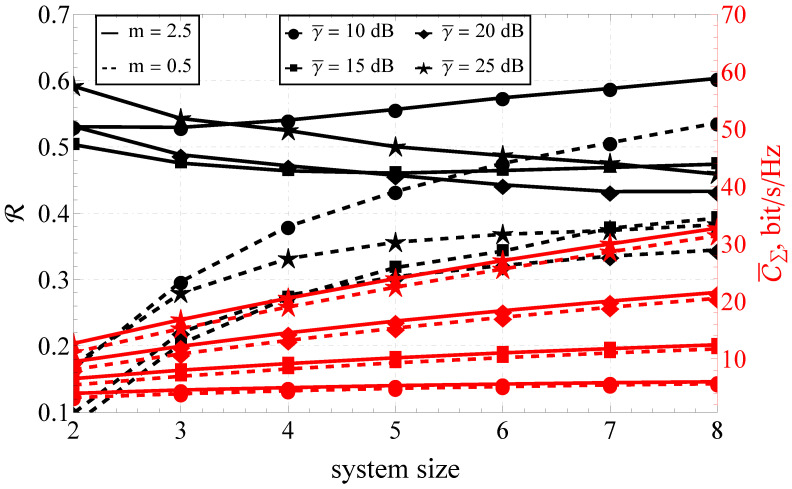
The joint R–${\bar{C}}_{\Sigma }$ performance of the variable-size MIMO system and different $\bar{\gamma }$’s.

Furthermore, the section is divided into the two following parts: capacity analysis and capacity reliability analysis. In Figure 1, Figure 2 and Figure 3, the results obtained with the help of the derived expressions (depicted with solid and dashed lines) are appended with the ones obtained via a Monte Carlo simulation (depicted by markers). For the reliability analysis in Section 4.2, the plots of the capacity reliability (obtained via numeric simulation) are supplied with a second vertical axis (see Figure 2, Figure 3, Figure 4, Figure 5, Figure 6, Figure 7 and Figure 8) depicting the capacity itself, thus delivering a joint study of the first- and second-order statistics.

### 4.1. Simulation Results for the Ergodic Capacity

Considering the MIMO system’s ergodic capacity $\bar{C}$ (see Figure 1) as a function of the one-step correlation coefficient ρ (for various channel parameters, i.e., Ω,m), one can see that its increase reduced $\bar{C}$, meaning that it degraded the communication quality.

The Nakagami parameter *m* had little overall effect on the dependencies under consideration. A sensible discrepancy between the curves (with the same ρ and different *m*’s) was observed at ρ≤0.3 and $\bar{\gamma }\geq 24$ dB. It can be seen that the derived expression for $\bar{C}$ (see (23)) had an excellent agreement with the simulation.

For small average signal-to-noise ratios (e.g., $\bar{\gamma }\leq 10$ dB), the channel parameters’ impact was vanishing. This generally means that in the low-SNR regime, there is no pronounced difference in the system performance between the hyper-Rayleigh and lighter-than-Rayleigh fading.

### 4.2. Simulation Results for the Capacity Reliability

As mentioned earlier, the main focus of the proposed research was on capacity reliability. To broaden and deepen the system’s functioning analysis and strengthen the derived conclusions, the second- and first-order capacity statistics were studied jointly by combining the graphs for R (i.e., capacity reliability) and ${\bar{C}}_{\Sigma }$ (i.e., the total ergodic capacity across all streams) in one plot (see Figure 2, Figure 3, Figure 4, Figure 5, Figure 6, Figure 7 and Figure 8).

The classical studies (ignoring the higher-order statistics) usually conclude that the increase of $\bar{\gamma }$ leads to the increase of ${\bar{C}}_{\Sigma }$ (see red lines in Figure 2). Nevertheless, for the same range of average SNR, R exhibits a pronounced minimum (see black lines in Figure 2), which implies the increase of the fluctuation range of instantaneous capacities. Thus, from a practical perspective, it is advised to avoid functioning in the vicinity of this extremum.

For example (see Figure 2), considering as an illustration the joint R–${\bar{C}}_{\Sigma }$ analysis of the 8×8 MIMO system with the received fluctuating signal average power Ω=1 and various one-step correlation coefficients ρ, several conclusions can be drawn:

- As expected, ${\bar{C}}_{\Sigma }$ increased monotonically over the entire interval of $\bar{\gamma }$.
- The increase of the correlation coefficient decreased ${\bar{C}}_{\Sigma }$.
- The extremum of the capacity reliability was attained at around 20 dB. The specific value of $\bar{\gamma }$ for ${\bar{C}}_{\Sigma_{\min}}$ depended on ρ.
- For $\bar{\gamma }\leq 20$ dB, R monotonically decreased, which negatively affected ${\bar{C}}_{\Sigma }$.
- The overall dependence of R from ρ for $\bar{\gamma }<20$ dB and $\bar{\gamma }>20$ dB was strictly the opposite. This meant that in the lower-SNR range, the increase of the antenna correlation actually improved the reliability; for the higher SNR, the increase of ρ impaired R.

An illustrative example, depicted in Figure 2, demonstrated the existence of a pronounced minimum of the capacity reliability and thus the necessity of a joint R–${\bar{C}}_{\Sigma }$ analysis, although it can be argued whether this effect could not have been removed by increasing the number of antenna elements (i.e., MIMO system size). Previously, for a SIMO system with a shadowed fading channel (described with a κ–μ shadowed model) [15], it was demonstrated that the increase of the transmitting/receiving antenna arrays did not eliminate the decrease of R for certain values of $\bar{\gamma }$.

To study that effect for the assumed problem, a numeric analysis and a simulation were performed. For this, the MIMO system size was extended to 32 elements (see Figure 3), and the fading parameters were set to m=1,Ω=1. The results were obtained with the help of the derived expressions (23) and (31) for a low ρ=0.1 and moderate ρ=0.5 correlation, depicted with solid and dashed lines, respectively. They were appended with the results obtained via the Monte Carlo simulation (depicted with markers).

The performed analysis made it possible to conclude that the increase of *N*, as it was expected, improved ${\bar{C}}_{\Sigma }$ (see Figure 3a)), and the increase of ρ impaired this effect (although at a different level, i.e., the greater the MIMO size, the stronger the impact of ρ). At the same time, it was interesting that *N* did not affect the minimum value of the capacity reliability $R_{min}$ but monotonically shifted the average SNR ${\bar{\gamma }}_{R_{min}}$ (where $R_{min}$ was attained) to the high-SNR region, which makes the joint R–${\bar{C}}_{\Sigma }$ analysis even more relevant for modern communication systems. Thus, since for given fading parameters, the $R(\bar{\gamma })$ curve’s form was independent of *N*, in the subsequent analysis, only the situations with N≤8 was assumed. Those cases corresponded to the existing communications standards.

Comparing the performance of 8×8 and 2×2 MIMO systems for a variable *m* (see Figure 4), it could be noted that the ordering of the curves varied depending on the average signal-to-noise ratio. This was confirmed by the presence of the above-mentioned extremum that existed at 20 dB in terms of reliability R in Figure 2.

It is worth mentioning that the reduction of the system size to 2×2 for certain signal-to-noise ratios led to the increase of the reliability. This meant that, in practice, R of the 2×2 system turned out to be higher than the one for the 8×8 system. This confirmed the importance of the higher-order statistics evaluation and illustrated that with certain channel parameters, increasing the number of antenna elements involved was impractical.

At the same time, the increase of the value of *m* did not induce sufficient changes in ${\bar{C}}_{\Sigma }$ if m≥1 for any number of elements, and R if m≥1.5 for the eight-element system. Therefore, for the 8×8 MIMO system, the values of m≥1.5 could be considered asymptotic. At the same time, the lower-dimensional system exhibited a nontrivial behavior with a pronounced maximum of the capacity reliability around m=2.

It should be mentioned that the information about the aforementioned asymptotic behavior is beneficial. In practice, the channel parameters’ inference has to be done on-the-fly, and the knowledge that the estimated value is in the asymptotic region can be used to reduce the inference-procedure complexity, leveraging the computational resources without loss of accuracy.

Analyzing the joint behavior of R–${\bar{C}}_{\Sigma }$ for different one-step correlation coefficients ρ (see Figure 5), one can observe that:

- For a small-dimensional system (e.g., 2×2), for any correlation coefficient, there was a noticeable extremum (maximum) of the capacity reliability. For a high-dimensional MIMO system (e.g., 8×8), R was a monotonically increasing function of *m*.
- The impact of the system correlation on R was negligible for $N_{T}=N_{R}\geq 6$.
- For the system size of 2×2 and the correlation coefficient ρ=0.5, the ergodic capacity ${\bar{C}}_{\Sigma }=8.08$ bits/s/Hz at m=2, and ${\bar{C}}_{\Sigma }=6.73$ bits/s/Hz at m=0.5. That is, 1.35 bits/s/Hz or a 16.7% loss (due to fading) of the maximum possible capacity. For the system size of 8×8 and the same correlation coefficient (i.e., ρ=0.5), the ergodic capacity ${\bar{C}}_{\Sigma }=15.48$ bits/s/Hz at m=2, and ${\bar{C}}_{\Sigma }=14.59$ bit/s/Hz at m=0.5, which meany that the loss equaled 0.89 bits/s/Hz or 5.75%.
- When considering the 2×2 system, the maximum ergodic capacity was attained at m=2. The decrease of the one-step correlation coefficient from 0.5 (maximally correlated system) to 0 (completely uncorrelated) increased the ergodic capacity from ${\bar{C}}_{\Sigma }=8.08$ bit/s/Hz to ${\bar{C}}_{\Sigma }=10.09$ bit/s/Hz, which equaled 2.01 bits/s/Hz or 19.92% of the maximum capacity. For the 8×8 MIMO system, functioning under the same conditions, a complete decorrelation increased ${\bar{C}}_{\Sigma }$ from 15.48 bits/s/Hz to 25.50 bits/s/Hz (i.e., 10.02 bits/s/Hz or 29.30% of the maximum capacity).

Thus, it can be concluded that for the small system sizes, the fading parameter *m* (in case of hyper-Rayleigh fading, i.e., m≤1) dominated the capacity reliability and significantly worsened the communication quality.

Comparing those results with the ones obtained for the intermediate number of antenna elements (see Figure 6), similar dependencies were observed (as for the 8×8 and 2×2 systems), but the effects were less pronounced. It is important to note that there was no extremum and the R–${\bar{C}}_{\Sigma }$ plots were monotonic.

Proceeding with the joint R–${\bar{C}}_{\Sigma }$ analysis for the variable system size (see Figure 7 and Figure 8), one can see the opposite behavior for the two extreme fading conditions. Increasing the number of antenna elements decreased the capacity reliability for the case of the light fading (i.e., m=2.5), and sufficiently increased it for the hyper-Rayleigh case (m=0.5). At the same time, the capacity itself increased almost linearly, justifying the supposition that the joint first- and second-order analysis yielded much more information about the system performance than the simple capacity analysis.

Moreover, expanding the size of the antenna system reduced the impact of correlation for lighter-than-Rayleigh fading and increased it for heavy fading. For the large system sizes, the effect of the fading was leveled (see Figure 7).

A similar analysis, applied to the varying $\bar{\gamma }$ (see Figure 8), led to analogous conclusions except for the case of light fading (m=2.5) and a small SNR ($\bar{\gamma }=10$ dB). In that scenario, the capacity reliability R behaved the same as in the case of heavy fading (m=0.5). This was explained by the presence of an extremum in $\bar{\gamma }$ for small system sizes (see Figure 2).

## 5. Conclusions

The presented research studied the problem of higher-order capacity description of the MU-MIMO system functioning in the presence of generalized multipath fading subjected to the complex Nakagami-m distribution. It was proposed to describe such a system with the help of a joint capacity/capacity reliability analysis, which was carried out under the assumptions of an existing correlation between the antenna elements, and zero-forcing postprocessing used for the user signal detection. Within the proposed framework, the closed-form expressions were derived for (a) the single-stream and sum-rate capacity moment-generating functions, (b) the zero-forcing multi-user MIMO ergodic capacity, (c) the capacity reliability and the amount of dispersion and (d) the general-order capacity statistics. A numerical verification of the derived expressions was performed, and it demonstrated an excellent correspondence with the simulation. A thorough joint analysis of the system performance was performed for all possible channel and system parameters and different fading scenarios: hyper-Rayleigh and lighter-than-Rayleigh fading. Several peculiarities of the system performance were observed and discussed. The performed research demonstrated the existence of a pronounced extremum of the capacity reliability for small-sized MIMO systems with respect to the fading Nakagami-m parameter, and the opposing behavior (depending on the system size) for heavy and light fading conditions. Specific values of the parameter, which were asymptotic for the ergodic capacity and capacity reliability, were identified.

## Figures and Tables

**Table 1:** System and channel parameters assumed for the simulation.

Parameter	Parameter Value
System size ($T_{x}$, $R_{x}$)	2×2…32×32
Decoding algorithm of the received signal	Zero-Forcing
Number of active users (i.e., active substreams, $N_{s}$)	2… 8
One–step correlation coefficient (ρ)	0…0.5
Fading parameter (*m*)	0.5…5
Average input signal-to-noise ratio for *k* substreams ($\bar{\gamma }$, dB)	0…50

## Data Availability

Not applicable.
